# Mechanisms of synergism between cisplatin and gemcitabine in ovarian and non-small-cell lung cancer cell lines

**DOI:** 10.1038/sj.bjc.6690452

**Published:** 1999-06

**Authors:** C J A van Moorsel, H M Pinedo, G Veerman, A M Bergman, C M Kuiper, J B Vermorken, W J F van der Vijgh, G J Peters

**Affiliations:** Department of Medical Oncology, University Hospital Vrije Universiteit, PO Box 7057, 1007 MB Amsterdam, The Netherlands

**Keywords:** cisplatin, gemcitabine, DNA damage, strand breaks, DNA adducts

## Abstract

2′,2′-Difluorodeoxycytidine (gemcitabine, dFdC) and *cis*-diammine-dichloroplatinum (cisplatin, CDDP) are active agents against ovarian cancer and non-small-cell lung cancer (NSCLC). CDDP acts by formation of platinum (Pt)–DNA adducts; dFdC by dFdCTP incorporation into DNA, subsequently leading to inhibition of exonuclease and DNA repair. Previously, synergism between both compounds was found in several human and murine cancer cell lines when cells were treated with these drugs in a constant ratio. In the present study we used different combinations of both drugs (one drug at its IC_25_ and the other in a concentration range) in the human ovarian cancer cell line A2780, its CDDP-resistant variant ADDP, its dFdC-resistant variant AG6000 and two NSCLC cell lines, H322 (human) and Lewis lung (LL) (murine). Cells were exposed for 4, 24 and 72 h with a total culture time of 96 h, and possible synergism was evaluated by median drug effect analysis by calculating a combination index (CI; CI < 1 indicates synergism). With CDDP at its IC_25_, the average CIs calculated at the IC_50_, IC_75_ IC_90_ and IC_95_ after 4, 24 and 72 h of exposure were < 1 for all cell lines, indicating synergism, except for the CI after 4 h exposure in the LL cell line which showed an additive effect. With dFdC at its IC_25_, the CIs for the combination with CDDP after 24 h were < 1 in all cell lines, except for the Cls after 4 h exposure in the LL and H322 cell lines which showed an additive effect. At 72 h exposure all Cls were < 1. CDDP did not significantly affect dFdCTP accumulation in all cell lines. CDDP increased dFdC incorporation into both DNA and RNA of the A2780 cell lines 33- and 79-fold (*P* < 0.01) respectively, and tended to increase the dFdC incorporation into RNA in all cell lines. In the AG6000 and LL cell lines, CDDP and dFdC induced > 25% more DNA strand breaks (DSB) than each drug alone; however, in the other cell lines no effect, or even a decrease in DSB, was observed. dFdC increased the cellular Pt accumulation after 24 h incubation only in the ADDP cell line. However, dFdC did enhance the Pt–DNA adduct formation in the A2780, AG6000, ADDP and LL cell lines (1.6-, 1.4-, 2.9- and 1.6-fold respectively). This increase in Pt–DNA adduct formation seems to be related to the incorporation of dFdC into DNA (*r* = 0.91). No increase in DNA platination was found in the H322 cell line. dFdC only increased Pt–DNA adduct retention in the A2780 and LL cell lines, but decreased the Pt–DNA adduct retention in the AG6000 cell line. In conclusion, the synergism between dFdC and CDDP appears to be mainly due to an increase in Pt–DNA adduct formation possibly related to changes in DNA due to dFdC incorporation into DNA. © 1999 Cancer Research Campaign


*British Journal of Cancer* (1999) **80**(7), 981–990
© 1999 Cancer Research Campaign
Article no. bjoc.1998.0452

### Mechanisms of synergism between cisplatin and gemcitabine in ovarian and non-small-cell lung cancer cell lines

**CJA van Moorsel, HM Pinedo, G Veerman, AM Bergman, CM Kuiper, JB Vermorken*, WJF van der Vijgh and GJ Peters**

Department of Medical Oncology, University Hospital Vrije Universiteit, PO Box 7057, 1007 MB Amsterdam, The Netherlands

**Summary** 2′,2′-Difluorodeoxycytidine (gemcitabine, dFdC) and *cis*-diammine-dichloroplatinum (cisplatin, CDDP) are active agents against ovarian cancer and non-small-cell lung cancer (NSCLC). CDDP acts by formation of platinum (Pt)–DNA adducts; dFdC by dFdCTP incorporation into DNA, subsequently leading to inhibition of exonuclease and DNA repair. Previously, synergism between both compounds was found in several human and murine cancer cell lines when cells were treated with these drugs in a constant ratio. In the present study we used different combinations of both drugs (one drug at its $IC_{25}$ and the other in a concentration range) in the human ovarian cancer cell line A2780, its CDDP-resistant variant ADDP, its dFdC-resistant variant AG6000 and two NSCLC cell lines, H322 (human) and Lewis lung (LL) (murine). Cells were exposed for 4, 24 and 72 h with a total culture time of 96 h, and possible synergism was evaluated by median drug effect analysis by calculating a combination index (CI; CI < 1 indicates synergism). With CDDP at its $IC_{25}$, the average CIs calculated at the $IC_{50}$, $IC_{75}$ $IC_{90}$ and $IC_{95}$ after 4, 24 and 72 h of exposure were < 1 for all cell lines, indicating synergism, except for the CI after 4 h exposure in the LL cell line which showed an additive effect. With dFdC at its $IC_{25}$, the CIs for the combination with CDDP after 24 h were < 1 in all cell lines, except for the Cls after 4 h exposure in the LL and H322 cell lines which showed an additive effect. At 72 h exposure all Cls were < 1. CDDP did not significantly affect dFdCTP accumulation in all cell lines. CDDP increased dFdC incorporation into both DNA and RNA of the A2780 cell lines 33- and 79-fold ($P < 0.01$) respectively, and tended to increase the dFdC incorporation into RNA in all cell lines. In the AG6000 and LL cell lines, CDDP and dFdC induced > 25% more DNA strand breaks (DSB) than each drug alone; however, in the other cell lines no effect, or even a decrease in DSB, was observed. dFdC increased the cellular Pt accumulation after 24 h incubation only in the ADDP cell line. However, dFdC did enhance the Pt–DNA adduct formation in the A2780, AG6000, ADDP and LL cell lines (1.6-, 1.4-, 2.9- and 1.6-fold respectively). This increase in Pt–DNA adduct formation seems to be related to the incorporation of dFdC into DNA ($r = 0.91$). No increase in DNA platination was found in the H322 cell line. dFdC only increased Pt–DNA adduct retention in the A2780 and LL cell lines, but decreased the Pt–DNA adduct retention in the AG6000 cell line. In conclusion, the synergism between dFdC and CDDP appears to be mainly due to an increase in Pt–DNA adduct formation possibly related to changes in DNA due to dFdC incorporation into DNA.

**Keywords:** cisplatin; gemcitabine; DNA damage; strand breaks; DNA adducts

*cis*-Diammine-dichloroplatinum (cisplatin, CDDP) is an established anticancer drug with activity in a variety of solid tumour types, including head and neck cancer (HNC), ovarian cancer and non-small-cell lung carcinoma (NSCLC). Its major disadvantage, however, is a relapse in most tumours after an initial response (Scanlon et al, 1991). CDDP is generally considered to exert its cytotoxic effect by binding to DNA, resulting in a number of different adducts (Sundquist et al, 1990). A tentative relationship between platinum–DNA adduct (Pt–DNA adduct) levels and anti-tumour response in cultured cells (Terheggen et al, 1990) and in patients has been postulated (Parker et al, 1991).

2′,2′-Difluorodeoxycytidine (gemcitabine, dFdC) is a deoxycytidine analogue (Hertel et al, 1988) with clinical activity against several solid tumours, such as ovarian cancer, NSCLC, HNC and pancreatic cancer (Van Moorsel et al, 1997). After entering the cell, dFdC is phosphorylated to its triphosphate (dFdCTP) which can be incorporated into DNA, followed by one more deoxynucleotide, after which DNA polymerization stops (Huang et al, 1991), which probably determines its cytotoxic effect. Besides this effect, dFdC is also capable of inhibiting ribonucleotide reductase (RR) (Heinemann et al, 1988), an enzyme with a key role in DNA repair mechanisms.

Because of the mechanisms of action and different side-effects, CDDP being nephrotoxic and dFdC being myelotoxic, combination of these drugs has been investigated. In in vitro and in vivo studies a synergistic effect of both drugs was found in both CDDP-resistant and non-resistant tumours and tumour cell lines (Braakhuis et al, 1995; Bergman et al, 1996). Several possible mechanisms could be responsible for this interaction; CDDP might influence dFdC metabolism at its activation site or at the DNA level, while dFdC might interact with the accumulation of CDDP, the extent or nature of DNA platination, or the process of DNA repair. Previously, we observed that the accumulation of dFdCTP in a human ovarian cancer cell line (A2780) was not influenced by CDDP, but CDDP did cause a decrease of 40% of dFdCTP pools in the A2780 CDDP-resistant variant, ADDP. DNA strand break (DSB) formation in A2780 cells was lower at simultaneous incubation of both drugs

*Received 8 July 1998*
*Revised 4 November 1998*
*Accepted 4 December 1998*

*Correspondence to:* GJ Peters

*Present address: Department of Oncology, University Hospital Antwerp, Belgium.

**Table 1** $IC_{50}$s for all cell lines after 4-, 24- and 72-h exposure to each drug alone

| Cell line | CDDP (μM) | | | dFdC (nM) | | |
|---|---|---|---|---|---|---|
| | 4 h | 24 h | 72 h | 4 h | 24 h | 72 h |
| A2780 | 2.9 ± 0.6 | 1.2 ± 0.5 | 2.0 ± 0.8 | 12.4 ± 2.7 | 4.3 ± 2.2 | 2.2 ± 1.0 |
| ADDP | 197 ± 82 | 63 ± 15 | 52 ± 13 | 239 ± 117 | 193 ± 43 | 625 ± 154 |
| AG6000 | 17.1 ± 4.5 | 4.7 ± 0.9 | 4.5 ± 1.0 | > $10^6$ | > $10^6$ | 50500 ± 20200 |
| H322 | 44.6 ± 9.2 | 15.3 ± 3.7 | 14.9 ± 2.7 | 708 ± 335 | 420 ± 201 | 120 ± 54 |
| LL | 7.5 ± 1.3 | 2.7 ± 0.7 | 2.9 ± 0.8 | 800 ± 100 | 27.3 ± 6.7 | 12.8 ± 4.4 |

The $IC_{50}$ is defined as the concentration causing 50% growth inhibition in treated cells when compared to control cells. Values are means ± s.e.m. of at least three separate experiments.

**Table 2** Evaluation of the interaction between dFdC and CDDP in ovarian and NSCLC cell lines by median drug effect analysis

| Cell line | Exposure time (h) | dFdC at approximate $IC_{25}$ CDDP variable | CDDP at approximate $IC_{25}$ dFdC variable |
|---|---|---|---|
| | | Average CI ± SEM | Average CI ± SEM |
| A2780 | 4 | 0.66 ± 0.17 | 0.45 ± 0.09 |
| | 24 | 0.49 ± 0.16 | 0.40 ± 0.06 |
| | 72 | 0.52 ± 0.10 | 0.41 ± 0.09 |
| ADDP | 4 | 0.16 ± 0.11 | 0.19 ± 0.05 |
| | 24 | 0.19 ± 0.08 | 0.40 ± 0.02 |
| | 72 | 0.28 ± 0.12 | 0.29 ± 0.08 |
| H322 | 4 | 0.58 ± 0.31 | 1.01 ± 0.50 |
| | 24 | 0.59 ± 0.08 | 0.68 ± 0.16 |
| | 72 | 0.43 ± 0.16 | 0.74 ± 0.26 |
| LL | 4 | 1.31 ± 0.32 | 1.10 ± 0.32 |
| | 24 | 0.46 ± 0.20 | 0.56 ± 0.20 |
| | 72 | 0.43 ± 0.16 | 0.35 ± 0.13 |

Values represent the average CIs (non-mutually exclusive) of the fractions affected (FA) of 0.5, 0.75, 0.90 and 0.95 of the combination of dFdC and CDDP and are means of at least three separate experiments. Cells were exposed to the combination in a non-constant ratio, either with CDDP at its approximate $IC_{25}$, or dFdC at its approximate $IC_{25}$ combined with a range of the other drug with exposure times of 4-, 24- or 72-h exposure followed by 68-, 48- or 0-h drug-free period. CI > 1 indicates antagonism, CI = 1 is additive and CI < 1 is synergism.

than with each compound alone (Bergman et al, 1996). It was therefore concluded that the synergistic interaction is possibly caused by an effect of dFdC on the cellular metabolism of CDDP. dFdC may inhibit DNA repair, leading to a decreased rate of repair of Pt–DNA adducts by the cancer cell.

The purpose of the present investigation was to elucidate possible mechanisms of synergism between dFdC and CDDP. Emphasis was on ovarian and NSCLC cells, since combinations of both compounds have led to increased response rates of up to 54% in NSCLC and 71% in ovarian cancer clinical studies (Steward et al, 1996; Abratt et al, 1997; Crino et al, 1997; Van Moorsel et al, 1997; Krakowski et al, 1998; Nogue et al, 1998).

We compared the effect of CDDP on accumulation of dFdCTP, dFdC incorporation into DNA and RNA, and the extent of DNA-DSB caused by the combination, with the effects of dFdC on the accumulation of CDDP and formation and retention of Pt–DNA adducts.

### MATERIALS AND METHODS

### Drugs and chemicals dFdC (Gemcitabine) and [5-$^3$H]-dFdC (16.7 Ci $mmol^{-1}$) were a kind gift of Eli Lilly Inc. (Indianapolis, IN, USA) and were solubilized with phosphate-buffered saline (PBS) to a concentration of 10 μM. CDDP (cisplatin) was obtained from Bristol-Myers Squibb (Woerden, The Netherlands) and solubilized with PBS to a concentration of 3 μM. Final dilutions of both drugs were made in culture medium. All other chemicals were of analytical grade and commercially available.

### Cell culture

The experiments were performed with five different cell lines, with two major histological subtypes. For human ovarian cancer, A2780 was the parental cell line (Lu et al, 1988; Ruiz van Haperen et al, 1994*a*), ADDP, the variant with induced resistance to CDDP (Lu et al, 1988), and AG6000, the variant with induced resistance to gemcitabine (Ruiz van Haperen et al, 1994*a*). The ADDP cell line was included as a model for CDDP resistance due to both a decreased accumulation and Pt-DNA adduct formation. The AG6000 cell line was included as a negative control since gemcitabine is not activated in this cell line. For NSCLC we used the human H322 cell line (subtype BAC, NCI), and the murine LL tumour cell line (kindly provided by Dr Lelieveld). The murine cell line was included because this line is relatively resistant to CDDP and gemcitabine, both in-vitro and in-vivo. Therefore, the cell line was also used in simultaneously ongoing animal experiments

 *© 1999 Cancer Research Campaign*

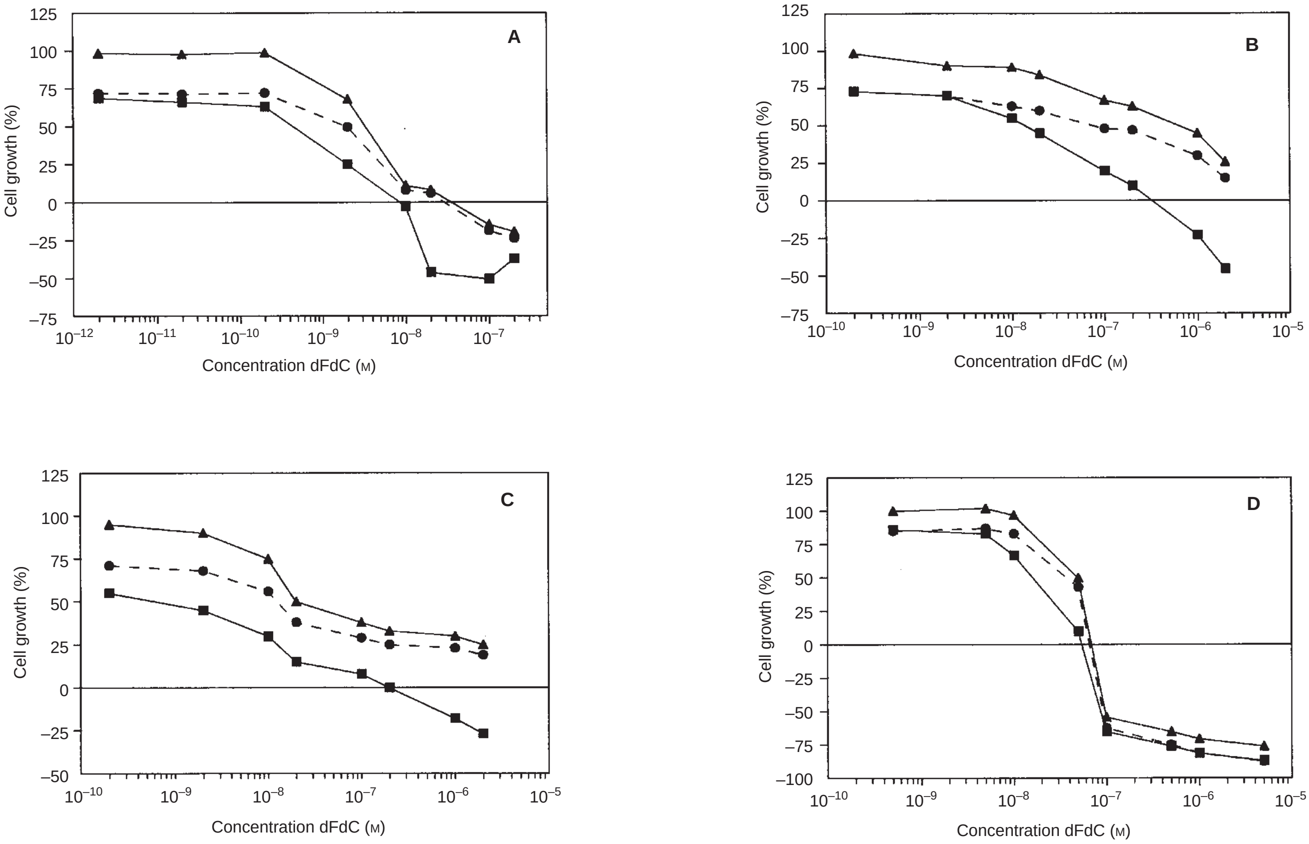

**Figure 1** Representative growth inhibition curves of the cell lines A2780 (**A**), ADDP (**B**), H322 (**C**) and LL (**D**). Cells were exposed to dFdC alone (▲) or in combination with CDDP at an $IC_{25}$ concentration (■) for 24 h. From the values of dFdC and CDDP alone the expected curve was calculated (●). After drug exposure, all cell lines were cultured in fresh medium. Total culture time was 72 h. All growth inhibition assays were repeated at least three times and the variation between experiments was always lower than 34%

(Van Moorsel et al, 1999). Doubling times of the cell lines were 21, 32, 37, 40 and 26 h respectively. A2780 and AG6000 cells were cultured in Dulbecco's medium with 5% heat-inactivated fetal calf serum (FCS). ADDP cells were cultured in RPMI medium with 5% heat-inactivated FCS. H322 and LL cells were cultured in RPMI medium with 10% heat-inactivated FCS. A total of 250 ng $ml^{-1}$ gentamicin was added to the media. All cell lines were growing exponentially as monolayers during the course of all experiments.

### Growth inhibition experiments

Growth inhibition experiments were performed in triplicate in 96-well flat-bottom plates (Costar, Cambridge, MA, USA) essentially as described previously (Peters et al, 1993*a*). Cells were seeded in 100-μl medium containing 5% FCS at different densities; 6000 per well for A2780 cells, 12 000 per well for ADDP cells, 20 000 per well for H322 cells and 5000 per well for LL cells. After 24 h, 100 μl of drug containing medium was added and cells were cultured for another 72 h. After 4 and 24 h the cells were washed and cultured in drug-free medium for 68 and 48 h respectively. Cells were exposed to dFdC alone or to CDDP alone, or to a combination of both drugs: one drug was added at a concentration causing about 25% growth inhibition, while the other drug was added at variable concentrations. The used CDDP concentrations in A2780, ADDP, H322 and LL cells for 4-h exposure were 6, 320, 88 and 2.7 μM respectively; for 24-h exposure 1.2, 160, 30 and 1.5 μM respectively; and for 72-h exposure 1.2, 120, 30 and 1.5 μM respectively. The used dFdC concentrations in A2780, ADDP, H322 and LL cells for 4-h exposure were 30, 320, 120 and 405 nM respectively; for 24-h exposure 4.8, 320, 40 and 10 nM respectively; and for 72-h exposure 4.8, 1400, 20 and 10 nM respectively. The CDDP concentration range for all cell lines was 10 nM to 0.5 μM, the dFdC concentration range was 0.02 nM to 1 μM in A2780 cells, 0.2 nM to 2 μM in ADDP and H322 cells and 5 nM to 10 μM in LL cells. Growth inhibitory effects were evaluated with the standard sulphorhodamine B protein (SRB) assay (Peters et al, 1993*a*). Growth of the cells was exponential during the whole incubation period. Relative growth was calculated as described previously (Monks et al, 1991; Peters et al, 1993*a*) by: $[(OD_{treated} - OD_{zero})/(OD_{control} - OD_{zero})] \times 100\%$, when $OD_{treated}$ was ≥ to $OD_{zero}$. In case $OD_{treated}$ was below $OD_{zero}$, cell killing had occurred. The optical density (OD) was read at 540 nm. The $OD_{zero}$ depicts the cell number at the moment of drug addition, the $OD_{control}$ reflects the cell number of untreated wells and the $OD_{treated}$ reflects the cell number in treated wells at the day of the assay.

*© 1999 Cancer Research Campaign* 

**Table 3** Effect of CDDP on the relative incorporation of dFdC into DNA and RNA of ovarian and lung cancer cells, corrected for inhibition of DNA and RNA synthesis

| | Ratio: dFdC incorporation into DNA / TdR incorporation into DNA | | | | Ratio: dFdC incorporation into RNA / UR incorporation into RNA | | | |
|---|---|---|---|---|---|---|---|---|
| Cell line | dFdC alone | | dFdC + CDDP | | dFdC alone | | dFdC + CDDP | |
| A2780 | 0.02 ± 0.01 | (95%) | 0.76 ± 0.16[a] | (99%) | 0.10 ± 0.02 | (0%) | 7.94 ± 0.88[a] | (87%) |
| ADDP | 0.05 ± 0.02 | (92%) | 0.03 ± 0.01 | (80%) | 0.12 ± 0.03 | (0%) | 0.64 ± 0.04 | (0%) |
| H322 | 0.30 ± 0.03 | (99%) | 0.07 ± 0.02 | (98%) | 0.85 ± 0.09 | (0%) | 1.53 ± 0.40 | (50%) |
| LL | 0.00 ± 0.00 | (76%) | 0.01 ± 0.00 | (71%) | 0.38 ± 0.07 | (0%) | 0.55 ± 0.14 | (0%) |

Cells were exposed to 0.1 μM dFdC alone, or in combination with 20 μM CDDP, for 24 h. Incorporation of $^3$H-dFdC into DNA and RNA was divided by the incorporation of TdR into DNA, and UR into RNA respectively. Values are means ± s.e.m. of three separate experiments (% DNA or RNA synthesis inhibition caused by the drug(s)). [a]Significantly different from dFdC alone ($P < 0.01$).

**Table 4** Effects of dFdC and CDDP on DNA strand break (DSB) formation in ovarian and lung cancer cells after 24-h exposure to both compounds alone or in combination

| Cell line | Concentration | | % DSB | | |
|---|---|---|---|---|---|
| | dFdC (nM) | CDDP (μM) | dFdC | CDDP | dFdC + CDDP |
| A2780 | 1.5 | 0.75 | –2.4 ± 5.8 | 10.7 ± 10.4 | –3.4 ± 8.2 |
| ADDP | 1.5 | 0.75 | 38.9 ± 9.2 | –4.2 ± 21.2 | 18.6 ± 24.0 |
| AG6000 | 1.5 | 0.75 | 23.8 ± 1.6 | –44.4 ± 30.5 | 6.8 ± 33.9 |
| H322 | 100 | 5 | 22.3 ± 5.4 | 9.7 ± 9.7 | –0.7 ± 2.3 |
| LL | 10 | 2 | –1.4 ± 23.2 | –14.0 ± 2.8 | 18.5 ± 2.9[a] |

Values (in % decrease of amount of double-stranded DNA in untreated cells) are means ± s.e.m. of three separate experiments. [a]$P = 0.04$; measured DSB to expected DSB (DSB of both drugs added together). Expected DSB formation: 8.3% in A2780, 34.7% in ADDP, –20.6% in AG6000, 32.0% in H322 and –15.4% in LL cells. For comparison data of Bergman et al (1996) on the A2780 cell line are included. Exposure of cells to 10 μM VP-16 for 1 h was always included as an internal control for the assay and gave the following extent of DSB formation: A2780: 25.7 ± 15.4, ADDP: 20.9 ± 10.8, AG6000: 15.0 ± 10.1, H322: 19.3 ± 7.4 and LL: 21.4 ± 5.5%. The actual levels of dsDNA in untreated cells at the end of the unwinding time were 86% in A2780, 96% in ADDP, 92% in AG6000, 87% in H322 and 49% in LL cells. These values were subsequently set at 100% to calculate the relative values.

We evaluated possible synergism using the median drug effect analysis method of Chou and Talalay (1983, 1994), processed by a computer program developed by Chou and Hayball (1996) and commercially available as CalcuSyn (Biosoft, Cambridge, UK). $D_m$ values ($IC_{50}$ values) are calculated by the program by extrapolation. For the separate drugs, the respective growth inhibition parameters, expressed as fraction affected (FA) (e.g. a FA of 0.25 is a growth inhibition of 25%) were introduced. The CI (combination index) was calculated by the formula: CI = $[(D)_1/(D_x)_1] + [(D)_2/(D_x)_2] + [a(D)_1(D)_2/(D_x)_1(D_x)_2]$. Where $a = 1$ for mutually non-exclusive drugs; $(D)_1$ and $(D)_2$ are the doses of the separate drugs and their combination; and $(D_x)_1$ and $(D_x)_2$ are the doses resulting in a growth inhibition of x%. These doses are calculated by the formula: $D = D_m[FA/(1 - FA)]^{1/m}$, where $D_m$ is the dose required to produce a 50% growth inhibition, $FA$ is the fraction affected, and $m$ is the slope of the median plot. Since CIs changed with FA, the averaged CIs at $IC_{50}$, $IC_{75}$, $IC_{90}$ and $IC_{95}$ were used. An average CI < 1 indicates synergism, > 1 indicates antagonism and an average CI of 1 indicates additivity.

### dFdCTP accumulation

The effect of CDDP on the accumulation of dFdCTP was studied by exposing $2–4 \times 10^5$ cells, in 6-well plates in duplicate, to dFdC (0.1 μM, 1 μM), or to dFdC and CDDP (20 μM, 200 μM) for 24 h. As a control, non-exposed cells were cultured for the same period. At the end of the incubation cells were washed in ice-cold PBS, harvested by rapid trypsinization (1 min at room temperature) and subsequently suspended in ice-cold culture medium with FCS, immediately followed by chilling on ice and cell counting. Nucleotides were extracted and analysed by HPLC as described previously (Bergman et al, 1996; Ruiz van Haperen et al, 1994*b*). Separation and quantification of the normal ribonucleotides and of dFdCTP was achieved with a gradient HPLC system (Partisphere SAX anion exchange column) connected to a photo-diode array detector, regularly set at 254 and 280 nm as described previously (Ruiz van Haperen et al, 1994*b*). Peaks were quantitated by a data acquisition program.

### [5-$^3$H]-dFdC incorporation

Incorporation of $^3$H-dFdC into DNA and RNA was performed essentially as described previously for measuring the incorporation of 5-fluorouracil (5-FU) into RNA and DNA and $^3$H-deoxyuridine into DNA (Peters et al, 1987; Van der Wilt et al, 1993) using 96-well filter-bottom plates (Multiscreen® Filtration System, 0.22 μm Hydrophilic Low Protein Binding Durapore® Membrane, Millipore, Molsheim, France). Briefly, cells (about 150 000 per well in 100 μl culturing medium) were plated and, after 24 h of recovery, incubated with [5-$^3$H]-dFdC (22 Ci mmol$^{-1}$) (0.1 and 0.4 μM) alone, or in combination with CDDP (20 and 200 μM) for 24 h at 37°C. The incubation was terminated by the addition of

 © 1999 Cancer Research Campaign

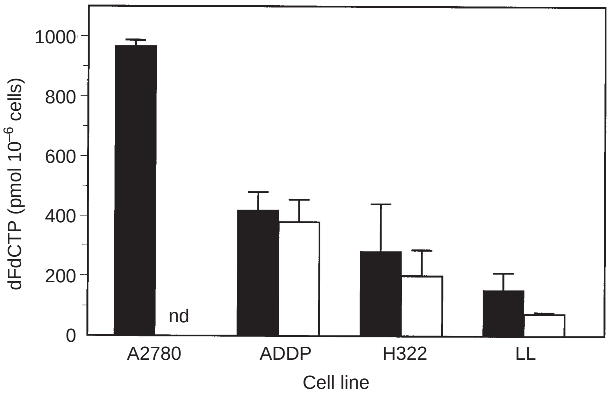

**Figure 2** Effect of CDDP on the accumulation of dFdCTP. For comparison, previously published effects of the A2780 cell line are also included (Bergman et al, 1996). Cells were exposed for 24 h to either 1 µM dFdC alone (black bars) or in combination with 200 µM CDDP (white bars). Values are means ± s.e.m. of three to five experiments. ND = not detectable, in A2780 because of cell death after combination of both compounds at these concentrations. AG6000 did not accumulate dFdCTP (Ruiz van Haperen et al, 1994)

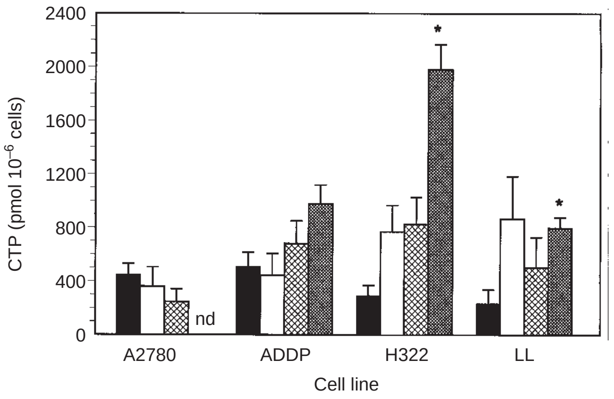

**Figure 3** CTP pools in the A2780, ADDP, H322 and LL cell lines in control cells (black bars), or after exposure of cells to 1 µM dFdC alone (white bars), 200 µM CDDP alone (crossed bars), or in combination (dense crossed bars) for 24 h. Values are means ± s.e.m. of three to five experiments. ND = not detectable, in A2780 because of cell death after combining both compounds at these concentrations. *Significantly different from control levels, $P < 0.05$

trichloroaretic acid (TCA) as described previously by Van der Wilt et al (1993). Incorporation into RNA was determined by adding 40 µl DNAase I (1 mg $ml^{-1}$) and 60 µl PBS to one part of the wells and an incubation for 30 min at 37°C. Incorporation into DNA was determined by adding 20 µl RNAase A/T1 (500 U $ml^{-1}$; DNAase-free) and 80 µl PBS to the other part of the wells and incubation for 30 min at 37°C. The reaction was terminated by precipitation of RNA and DNA, respectively, with TCA. Filters were washed with water and ethanol and, subsequently, the filters were incubated with NaOH to hydrolyse nucleic acids and counted. To determine the amount of cells, duplicate cultures were exposed to similar concentrations of non-radiolabelled dFdC, harvested by rapid trypsinization and counted. $^{3}$H-dFdC incorporation into DNA and RNA was corrected for inhibition of DNA and RNA synthesis, as measured by the incorporation of [2-$^{14}$C]-thymidine ($^{14}$C-TdR, 62.8 Ci $mol^{-1}$, 2.5 µM) and [5- $^{3}$H]-uridine ($^{3}$H-UR, 27 Ci $mmol^{-1}$, 58.6 nM) added to control cells and cells exposed for 24 h to dFdC and CDDP, respectively, 2 h before the end of the incubation, using a similar protocol as described before (Ruiz van Haperen et al, 1993). A ratio was calculated between dFdC incorporation into DNA and RNA, and TdR and UR incorporation into DNA and RNA, respectively, as follows:

DNA: (fmol dFdC in DNA $10^{-6}$ cells)/(fmol TdR in DNA $10^{-6}$ cells)
RNA: (fmol dFdC in RNA $10^{-6}$ cells)/(fmol UR in RNA $10^{-6}$ cells)

### FADU DNA-damage assay

The extent of DNA strand breaks (DSB) caused by dFdC and dFdC in combination with CDDP were measured by the FADU assay (Fluorometric Analysis of DNA Unwinding) as described previously by Birnboim and Jevcak (1981) and slightly modified (Bergman et al, 1996; Van der Wilt, 1997). This assay is based on the principle that the rate of unwinding of DNA under alkaline conditions depends on the presence of strand breaks; DNA with a high amount of strand breaks will unwind faster under alkaline conditions than DNA with no strand breaks. Double-stranded DNA (dsDNA) can be detected by ethidium bromide (EtBr) staining. Cells (about 5 × $10^{6}$ cells) were incubated with CDDP alone (ADDP and AG6000: 750 nM; H322: 5 µM; LL: 2.5 µM) or in combination with dFdC (ADDP and AG6000: 1.5 nM; H322: 100 nM; LL: 10 nM) for 24 h at 37°C. Etoposide (VP-16) was used as a positive control drug, and added at 50 µM to the cells 1 h before harvesting. Untreated cells were used as controls. Cells were harvested, kept on ice and directly used in the assay. For this purpose the cells were suspended in 2 ml ice-cold 0.25 M mesoinositol, 10 mM $NaH_2PO_4$, 1 mM magnesium chloride (pH 7.2) and the suspension was divided equally among three sets of tubes: T-, B- and P-tubes. All tubes were incubated with a buffer containing a high concentration of urea to disrupt the chromatin. T-tubes (total fluorescence) were then treated with glucose containing buffer, to stabilize DNA so that unwinding could not occur due to the alkaline environment. Subsequently, alkaline buffer was added. B-tubes (background fluorescence) were vortexed vigorously so that the dsDNA is sheared. All tubes were incubated at 15°C so that the DNA could unwind and were then put on ice. The glucose-containing buffer was then added to the P-tubes (estimate of unwinding rate of the DNA caused by the drug) and B-tubes. EtBr was added to all tubes and all tubes were vortexed. The fluorescence was measured and the extent of DNA strand breaks was calculated by: $(P - B)/(T - B) \times 100\%$.

### Total cellular platinum accumulation

Cells (about 5 × $10^{6}$ cells) were incubated with CDDP alone (20 and 200 µM), or in combination with dFdC (0.1 and 1 µM) for 24 h at 37°C. Cells were trypsinized, washed three times with ice-cold PBS, harvested, counted and stored as pellets at –20°C. Before analysis, 500 µl benzethonium hydroxide (hyamine) per 1 × $10^{6}$ cells was added to the cell pellets. A total of 25 µl water was added to the samples and standard curves were made by addition of 25 µl of standard CDDP-solutions (0.1–0.3 µM) in water to 1 × $10^{6}$ non-treated cells; all samples were vortexed and incubated overnight at 55°C; thereafter 4.25 ml 0.2 M hydrochloric acid was added. Samples were analysed on a Varian SpectrAA-10 atomic absorption spectrometer (Varian UK, Walton-on-Thames, Surrey, UK) equipped with a graphite furnace; data were formatted and archived on a personal computer utilizing Varian Report Manager software. Samples were dried at 95–110°C, ashing was performed at 1300°C and atomization at 2600°C.

© 1999 Cancer Research Campaign

### Platinum–DNA adduct determination

Cells were treated with CDDP (20 and 200 μM) alone or in combination with dFdC (0.1 and 1 μM) for 24 h at 37°C. After this time period drugs were washed away and cells were cultured in drug-free medium for another 3, 6 or 24 h. Cells were washed with PBS, trypsinized and harvested on ice; the cell pellets (about $5 \times 10^6$ cells) were resuspended in 1.0 ml lysis-buffer (100 mM Tris, 5 mM EDTA, 0.2% sodium dodecyl sulphate, 200 mM sodium chloride, 100 mg $ml^{-1}$ proteinase K, pH 8.5) and incubated for 2 days at 37°C with agitation. DNA was precipitated by mixing with 2-propanol and dissolved in TE-buffer (10 mM Tris, 1 mM EDTA, pH 7.5). DNA content was estimated by measuring optical density at 260 and 280 nm (protein content), all samples had an $OD_{260}/OD_{280}$ ratio > 1.9 indicating uncontaminated DNA. A total of 0.1 volume sodium chloride (1.65 M) was added to the dissolved DNA. A calibration curve was made using different solutions of CDDP (0–1.5 μM) in TE-buffer containing 0.165 M sodium chloride. Pt content of samples and standards was measured using AAS.

### Statistical evaluation

Results were evaluated using the paired and unpaired Student's *t*-test. Relations between parameters were evaluated using the Pearson's correlation test.

### RESULTS

### Analysis of the interaction between dFdC and CDDP

The $IC_{50}$s of dFdC and CDDP alone in the A2780, ADDP, AG6000, H322 and LL cell lines are summarized in Table 1. Clear differences were observed in the sensitivity for both drugs in these cell lines: at all exposure times A2780 is the most sensitive cell line for both compounds, followed by LL. The ADDP, AG6000 and H322 cell lines all are very resistant to dFdC (> 50-fold compared to A2780). ADDP is the most resistant cell line to CDDP (> 45-fold resistant), followed by H322 (> 7.5-fold resistant) and AG6000 (> 3.5-fold resistant). Based on these sensitivity data, combination experiments were designed in which cells were exposed to the approximate $IC_{25}$ of one drug and a concentration range of the other drug. From the separate growth inhibition data, expected curves could be calculated. Figure 1 shows representative growth inhibition curves for dFdC alone, the combination of dFdC and CDDP, and the expected growth inhibition curves in the A2780, ADDP, H322 and LL cell lines. It was remarkable that in the A2780, ADDP and H322 cell lines the highest difference between the expected and measured curve was observed at the $IC_{100}$ concentration of dFdC.

Synergism was analysed with the median drug effect analysis of Chou and Talalay (1983, 1994), average CIs of the FA 0.5, 0.75, 0.90 and 0.95 are given in Table 2. At 4-h exposure of cells to the approximate $IC_{25}$ of dFdC in combination with CDDP synergism was found in the A2780, ADDP and H322 cell lines. However, slight antagonism was found in the LL cell line. At 24- and 72-h exposures synergism was found in all cell lines. At 4-h exposure to CDDP at the approximate $IC_{25}$ and to dFdC in a concentration range, the combination was synergistic in the two ovarian cancer cell lines A2780 and ADDP. However, additivity was found in the H322 cell line and moderate antagonism in the LL cell line. At 24- and 72-h exposures synergism was found in all cell lines.

### Effects on dFdCTP accumulation and normal nucleotide pools

In order to determine a possible role of dFdCTP in the interaction between dFdC and CDDP, we measured the accumulation of dFdCTP after 24-h exposure to 1 μM dFdC alone, or in combination with 200 μM CDDP in the A2780, ADDP, H322 and LL cell lines (Figure 2). dFdCTP accumulation after exposure to 1 μM dFdC alone for 24 h did not show a clear relation with the sensitivity of this panel of cell lines. A2780 cells, which are the most sensitive to dFdC, clearly accumulated the highest amount of

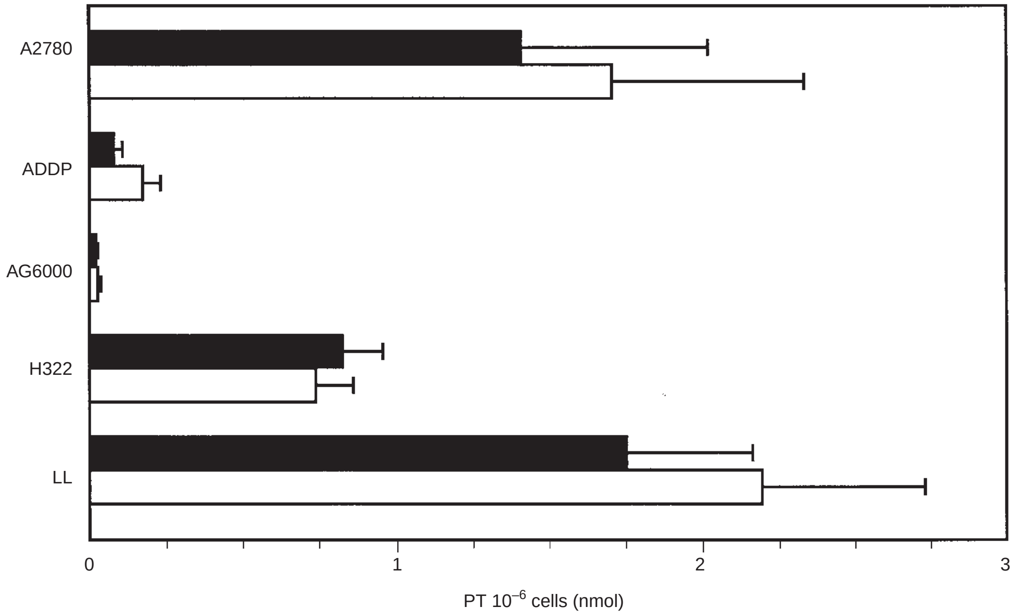

**Figure 4** Effect of dFdC on the cellular accumulation of platinum in ovarian and lung cancer cell lines. Cells were exposed to either CDDP alone (200 μM) (■) or in combination with dFdC (1 μM) (□) for 24 h. Values are means ± s.e.m. of three to four experiments

 © 1999 Cancer Research Campaign

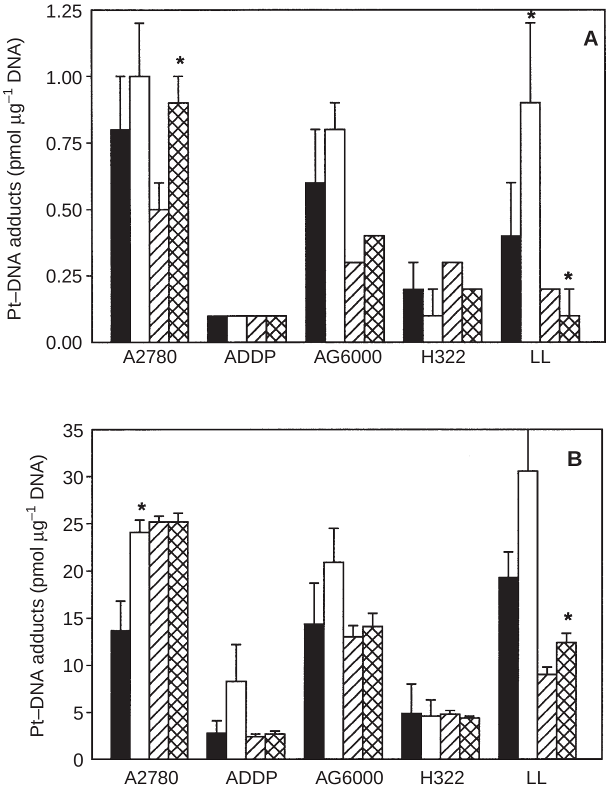

**Figure 5** Pt–DNA adduct levels in DNA after 24-h exposure to CDDP alone (solid bars) or CDDP and dFdC in combination (open bars), and 3-h incubation in drug-free medium (hatched bars: CDDP alone; double hatched bars: CDDP and dFdC in combination. (**A**) Results of 20 µM CDDP and 0.1 µM dFdC. (**B**) Results of 200 µM CDDP and 1 µM dFdC. This Figure shows the mean data of at least three experiments ± s.e.m. *Significantly different from CDDP alone, $P < 0.05$

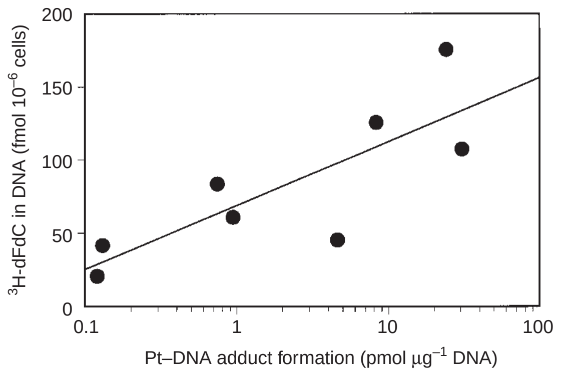

**Figure 6** Correlation between $^{3}$H-dFdC incorporation into DNA (data not shown) and initial Pt–DNA adduct formation (Figure 5) in the A2780, ADDP, H322 and LL cell lines. Pearson correlation $r = 0.91$. $P$-value (two-tailed) = 0.02

dFdCTP. However, LL cells, which are the second most sensitive to dFdC, accumulate the lowest amount of dFdCTP. CDDP did not cause any significant changes in dFdCTP accumulation, even though it tended to decrease dFdCTP accumulation 10, 25 and 50% in the ADDP, H322 and LL cell lines respectively. In A2780 cells, the combination of both compounds at these concentrations was too toxic for reliable measurements of the dFdCTP accumulation. Normal nucleotide pools were evaluated in the same analysis; only CTP pools showed relevant changes and are shown in Figure 3. Incubation with 1 µM dFdC alone tended to increase CTP pools 2.6- and 3.7-fold in the H322 and LL cell lines ($P = 0.09$ and $P = 0.13$) respectively, whereas no differences were found in the A2780 and ADDP cell lines. CDDP alone also tended to increase CTP pools in the H322 line 2.8-fold ($P = 0.07$). However, the combination of both compounds resulted in a significant increase of CTP pools in the ADDP, H322 and LL cells (1.9-, 6.7- and 3.4-fold; $P = 0.06$, $P < 0.01$ and $P = 0.03$ respectively).

### dFdC incorporation into DNA and RNA

To determine the possible contribution of dFdC incorporation into DNA and RNA to the interaction between both compounds, incorporation of [5-$^{3}$H]-dFdC into DNA and RNA was studied. Within the panel of human cell lines the most sensitive cell line to dFdC, A2780, incorporated threefold more dFdC into DNA ($P = 0.02$ and $P = 0.01$ respectively) and fivefold more dFdC into RNA than the more resistant cell lines ADDP and H322 respectively ($P < 0.01$ for both cell lines) (results not shown). However, the A2780 cell line incorporated threefold less dFdC into DNA than the less-sensitive murine LL cell line ($P = 0.02$). The amount of dFdC incorporation into DNA did not show a clear relation with the dFdCTP accumulation in these cell lines. The LL cell line accumulated the lowest amount of dFdCTP; however, it incorporated the highest amounts of dFdC into both DNA and RNA. Together this possibly resulted in the rather sensitive phenotype.

The effects of CDDP on dFdC incorporation into DNA and RNA were corrected for the incorporation of TdR and UR into DNA and RNA as a parameter for DNA and RNA synthesis (Table 3). Using this correction, CDDP increased dFdC incorporation into both DNA and RNA of the A2780 cell line 33- and 79-fold ($P < 0.01$) respectively, and did not influence the incorporation of dFdC into DNA in the other cell lines. CDDP tended to increase the dFdC incorporation into RNA in all cell lines. For the high drug concentrations (both dFdC and CDDP), DNA and RNA synthesis were completely inhibited and no reliable ratio could be calculated.

### DSB formation

The extent of DSB formation after exposure to either CDDP or dFdC alone, or to a combination of both compounds, was measured to determine the possible contribution of this type of DNA damage to the interaction between both compounds (Table 4). Expected values were calculated by addition of the amount of DSB formed by each compound alone. In the wild-type ovarian cancer cell line A2780, in the CDDP-resistant ADDP cells and in the NSCLC H322 cells less DSB than expected tended to be formed by the combination of dFdC and CDDP (differences: 11.7%, 16.1% and 32.7% respectively). However, in the dFdC-resistant AG6000 cells, and in the murine LL cells, more DSB than expected were formed (27.4% and 33.9% respectively; not significant in AG6000 cells; $P = 0.04$ in LL cells).

### Total cellular platinum accumulation

The amount of total Pt accumulating in cells after exposure of cells to CDDP alone, or in combination with dFdC, was determined to study whether dFdC would affect total cellular Pt accumulation. Figure 4 shows the Pt accumulation in the ovarian cancer and NSCLC cell lines after 24 h of incubation with either CDDP alone,

*© 1999 Cancer Research Campaign*

or in combination with dFdC. In the A2780 cell line, exposure to 200 μM CDDP resulted in 60- and 17-fold higher Pt accumulation than in the CDDP-resistant variants AG6000 and ADDP respectively ($P < 0.05$). The NSCLC cell lines, H322 and LL, both accumulated more Pt than the resistant ovarian cancer cell lines. Total Pt accumulation in this panel apparently is not related to CDDP sensitivity. A significant effect of dFdC on Pt accumulation was only found in the ADDP cell line; dFdC caused a 2.1-fold increase of Pt accumulation in this cell line ($P = 0.04$).

### Pt–DNA adduct formation

Formation of Pt–DNA adducts is a critical event in the cytotoxicity of CDDP. Therefore, we studied whether dFdC might affect the formation of Pt–DNA adducts and the Pt–DNA adduct retention (Figure 5A,B). The CDDP-resistant cell line ADDP clearly formed 4.9-fold fewer Pt–DNA adducts compared to its sensitive parental cell line A2780 ($P = 0.02$), which may be related to its lower total cell Pt accumulation. This was in contrast to the Pt–DNA adduct formation in the AG6000 cell line, which was similar to that in the A2780 cell line, although Pt accumulation in the AG6000 cells was much lower than in A2780 cells. The Pt–DNA adduct formation in the LL lung cancer cell line was fourfold higher than that in the H322 cells ($P < 0.01$), which was in line with the higher Pt accumulation in these cells. dFdC increased the Pt–DNA adduct formation compared to CDDP alone in all cell lines except in H322 and ADDP cells at the low concentrations. However, in the ADDP cell line, adduct levels were at the detection limit of the atomic absorption spectroscopy. At the high CDDP concentration, dFdC increased the Pt–DNA adduct formation in ADDP cells almost to the level found in A2780 cells treated with CDDP alone. The level of Pt–DNA adduct formation correlated with the incorporation of dFdC into DNA after 24 h exposure to dFdC alone (Figure 6).

The retention of the Pt–DNA adducts formed after exposure to 200 μM CDDP was increased similarly at 3 (Figure 5B), 6 and 24 h (data not shown) by co-exposure to 1 μM dFdC in the LL cell line (1.4-, 1.3- and 1.3-fold; $P < 0.01$, $P < 0.18$ and $P < 0.19$ respectively). However, in the dFdC-resistant AG6000 cell line, the 6- and 24-h Pt–DNA adduct levels in cells after treatment with the combination of dFdC and CDDP were 85% of the levels in cells treated with CDDP alone ($P = 0.10$ and $P < 0.01$ respectively) (data not shown). In all other cell lines no effect of dFdC on the 3-, 6- and 24-h retention of Pt–DNA was found. However, in most cell lines the level of Pt–DNA adducts 24 h after exposure to CDDP did not decrease significantly compared to the 3- and 6-h levels. Only in the AG6000 cells did Pt–DNA adduct levels decrease to about 50% of the initial levels (data not shown). It is possible that cells lacked an intact enzyme system due to these high concentrations, and thus were inhibited in their ability to repair DNA damage.

Therefore, we focused on the combination of 20 μM CDDP and 0.1 μM dFdC (Figure 5A). dFdC significantly increased the retention of Pt–DNA adducts in the A2780 cell line ($P < 0.05$). However, this effect did not last longer than 3 h and seemed to be due to the initial increase in Pt–DNA adduct levels rather than to DNA repair inhibition. In the LL cell line, dFdC caused a twofold decrease in Pt–DNA adducts after exposure to the low concentration of CDDP ($P = 0.02$). However, note that the Pt–DNA adduct levels after exposure to 20 μM CDDP were just above the detection level. In the AG6000, ADDP and H322 cell lines, dFdC did not seem to affect Pt–DNA adduct retention 3, 6 and 24 h after exposure.

When evaluated as total exposure to Pt–DNA adducts, in all cell lines except for the H322, the areas under the curve for Pt–DNA adduct levels tended to be higher for the dFdC–CDDP combination (a 1.5-, 2.2-, 1.4- and 1.8-fold increase in the A2780, ADDP, AG6000 and LL cell lines respectively (data not shown).

### DISCUSSION

In this study we showed synergism between dFdC and CDDP in several ovarian cancer and NSCLC cell lines. The most pronounced effects were found in the CDDP-resistant cell lines when CDDP was used around its $IC_{25}$ and dFdC in a concentration range. The mechanism of this synergistic interaction is most likely due to an increased Pt–DNA adduct formation, possibly related to the incorporation of dFdC into DNA.

Using a different approach of drug exposure than in previous studies, synergism was found in a panel of five different cell lines. It was remarkable that in some cell lines the best effect was observed at the $IC_{100}$ concentration of dFdC, indicating that the combination can exert significant anti-tumour activity by killing cells. The concentrations used in this study to achieve synergism are in agreement with levels of both drugs that can be reached in patients (Vermorken et al, 1984; Abbruzzese et al, 1991; Peters et al, 1993*a*; Freeman et al, 1995; Van der Uijgh, 1991). Combination of both compounds has led to increased response rates in various cancer types, such as ovarian cancer and NSCLC, in which response rates up to 71% were observed (Steward et al, 1996; Abratt et al, 1997; Crino et al, 1997; Van Moorsel et al, 1997; Krakowski et al, 1998; Nogue et al, 1998).

The present studies were performed to elucidate the mechanism of the interaction between dFdC and CDDP; therefore, various parameters related to the mechanism of action of both compounds were investigated. dFdCTP accumulation was related to sensitivity to dFdC of all cell lines tested in this study and in previous studies (Ruiz van Haperen et al, 1994*b*; Bergman et al, 1996) (except for the murine LL cells). CDDP did not cause any significant changes in dFdCTP accumulation, but tended to decrease the dFdCTP accumulation in the ADDP, H322 and LL cell lines. This phenomenon might be the result of the highly toxic combination of both compounds. However, since this decrease in dFdCTP accumulation was seen in dFdC- and CDDP-sensitive, as well as -resistant, cell lines in this study and in a previous study (Bergman et al, 1996), a more likely possibility is the rise in CTP and UTP pools, caused by both CDDP and dFdC. Both CTP and UTP can moderately inhibit the activity of dCK in competition with ATP (Ruiz van Haperen et al, 1996), therefore a rise in CTP and UTP might decrease the accumulation of dFdCTP. However, CDDP might also inhibit dFdC uptake of cells directly, which was already shown for 2′-deoxy-5-azacytidine (DAC), another deoxycytidine analogue (Ellerhorst et al, 1993).

In this study, no relation was found between dFdC incorporation into DNA and sensitivity to dFdC. The higher dFdC incorporation into DNA in LL cells than in the more dFdC-sensitive A2780 cell line might be due to the higher inhibition of DNA synthesis in A2780 cells, since this difference disappears after correction of the incorporation of dFdC for the inhibition of DNA synthesis. In the A2780 cell line, CDDP increased the incorporation of dFdC into DNA, possibly due to the inhibition of RR by both CDDP and dFdC (Heinemann et al, 1988; Chiu et al, 1992). Further research is warranted to study the mechanism responsible for the increase in dFdC incorporation into RNA and DNA by CDDP.

To study the possible interaction of both compounds with respect to DNA damage, we determined the effect of both drugs on

*© 1999 Cancer Research Campaign*

DNA integrity. The only cell lines in which dFdC did not cause any damage were the ovarian cancer cell line A2780 and the murine cell line LL, whereas CDDP caused DNA damage only in A2780 and H322 cells. CDDP even seemed to have a protective effect on DSB (values < 0%) in AG6000 and LL cells, which might be caused by the interstrand cross-links formed by this compound. In a previous study by Bergman et al (1996) it was postulated that dFdC incorporation into DNA could result in inhibition of the repair of Pt–DNA adducts, causing apparent stabilization of DNA. In the present study, this effect was indeed found in the A2780, ADDP and H322 cells, where a trend of less DSB formation than expected was found. However, in dFdC-resistant AG6000 cells more DSB than expected were formed, indicating that dFdC is necessary for the increase in DNA stabilization. An increase in DSB were also found in the murine cell line LL. Whether this phenomenon in the LL cell line is of much importance for the human situation remains to be seen. However, the above mechanisms apparently are not the only explanation for the synergistic interaction between both compounds in these cell lines.

dFdC hardly affected cellular Pt accumulation. The only exception was the ADDP cell line, in which dFdC clearly increased the Pt accumulation. In the cell CDDP binds to both DNA and protein, but to a much larger extent to protein (99%); thus the increases in Pt accumulation in ADDP cells (overall 30%) cannot be explained by the increase in Pt–DNA adduct formation. Therefore, an effect of dFdC on the binding of CDDP to intracellular proteins cannot be excluded.

The increase in Pt–DNA adduct formation could be the result of several effects of dFdC at the DNA level. Studies on the mechanism of interaction between DAC and CDDP revealed an increase of Pt–DNA binding on DAC substituted plasmid DNA (Abbruzzese and Frost, 1992), which was not hypomethylation dependent. Similarly, in our study there was a correlation between the incorporation of dFdC into DNA and the initial Pt–DNA adduct formation. The incorporation of dFdC into DNA could lead to structural changes favouring the binding of Pt to the guanine nucleotide opposite to the cytosine nucleotide and thus be of major importance in the synergistic interaction of both compounds.

Since the retention of DNA platination was not increased in the cell lines studied, dFdC did not seem to affect the overall DNA repair of Pt–DNA adducts in this setting. However, since the initial rapid repair of Pt–DNA adducts already starts a few hours after the adduct formation, this effect could in this study have been masked by the prolonged exposure period we used to be in accordance with the growth inhibition experiments.

Strikingly, the largest increase in initial Pt–DNA adduct formation by dFdC was found in the CDDP-resistant ADDP cell line, although this cell line did not show a significant increase in DNA stabilization after exposure to CDDP combined with dFdC. dFdC might favour the formation of intra- instead of interstrand Pt cross-links in this cell line. No increase in formation and retention of Pt–DNA adducts was found in the H322 cell line. Since this cell line had the lowest level of dFdC incorporation into DNA, it might be postulated that a certain threshold dFdC incorporation is needed in a cell to induce the increased Pt–DNA adduct formation.

This study shows that the combination of dFdC and CDDP can be synergistic in various cancer cell lines with a different histologic origin. The mechanism of this synergy is most likely an increase in the formation of Pt–DNA adducts, possibly related to the incorporation of dFdC.

### ACKNOWLEDGEMENTS

This study was supportal by grant IKA-VU 94–753 from the Dutch Cancer Society.

### REFERENCES

Abbruzzese JL and Frost P (1992) Studies on the mechanism of the synergistic interaction between 2′-deoxy-5-azacytidine and cisplatin. *Cancer Chem Pharmacol* **30**: 31–36

Abbruzzese JL, Grunawald R, Weeks EA, Gravel D, Adams T, Nowak B, Mineishi S, Tarassoff P, Satterlee W, Raber MN and Plunkett W (1991) A phase I clinical, plasma, and cellular pharmacology study of gemcitabine. *J Clin Oncol* **9**: 491–498

Abratt RP, Bezwoda WR, Goedhals L and Hacking DJ (1997) Weekly gemcitabine with monthly cisplatin: effective chemotherapy for advanced non-small cell lung cancer. *J Clin Oncol* **15**: 744–749

Bergman AM, Ruiz van Haperen VWT, Veerman G, Kuiper CM and Peters GJ (1996) Interaction between cisplatin and gemcitabine in vitro. *Clin Cancer Res* **2**: 521–530

Birnboim HC and Jevcak JJ (1981) Fluorometric method for rapid detection of DNA strand breaks in human white blood cells produced by low doses of radiation. *Cancer Res* **41**: 1889–1892

Braakhuis BJM, Ruiz van Haperen VWT, Welters MJP and Peters GJ (1995) Schedule-dependent therapeutic efficacy of the combination of gemcitabine and cisplatin in head and neck cancer xenografts. *Eur J Cancer* **31A**: 1335–1340

Chiu CSM, Chan AK and Wright JA (1992) Inhibition of mammalian ribonucleotide reductase by *cis*-diamminedichloroplatinum(II). *Biochem Cell Biol* **70**: 1332–1338.

Chou T-C and Hayball MP (1996) *CalcuSyn, Windows Software for Dose Effect Analysis*. Biosoft: Cambridge

Chou T-C and Talalay P (1983) Quantitative analysis of dose-effect relationship: the combined effects of multiple drugs on enzyme inhibitors. In *Advances in Enzyme Regulation*, G. Weber (ed), pp. 27–55. Pergamon Press: New York

Chou T-C, Motzer RJ, Tong Y and Bosl GJ (1994) Computerized quantitation of synergism and antagonism of taxol, topotecan, and cisplatin against human teratocarcinoma cell growth: a rational approach to clinical protocol design. *J Natl Cancer Inst* **86**: 1517–1524

Crino L, Scagliotto G, Marangolo M, Figoli F, Clerici M, DeMarinis F, Salvati F, Cruciani G, Dagliotti L, Pucci F, Paccagnella A, Adamao V, Altavilla G, Incoronato P, Tripetti M, Mosconi AM, Santucci A, Sorbolini S, Oliva C and Tonato M (1997) Cisplatin-gemcitabine combination in advanced non-small cell lung cancer: a phase II study. *J Clin Oncol* **15**: 297–303

Ellerhorst JA, Frost P, Abbruzzese JL, Newman RA and Chernajovsky Y (1993) 2′-deoxy-5-azacytidine increases binding of cisplatin to DNA by a mechanism independent of DNA hypomethylation. *Br J Cancer* **67**: 209–215

Freeman KB, Anliker S, Hamilton M, Osborne D, Dhahir PH, Nelson R and Allerheiligen SRB (1995) Validated assays for the determination of gemcitabine in human plasma and urine using high-performance liquid-chromatography with ultraviolet detection. *J Chromatogr Biomed Appl* **665**: 171–181

Heinemann V, Hertel LW, Grindey GB and Plunkett W (1988) Comparison of the cellular pharmacokinetics and toxicity of 2′,2′-difluorodeoxycytidine and 1-β-D-arabinofuranosylcytosine. *Cancer Res* **48**: 4024–4031

Hertel LW, Kroin JS, Misner JW and Tustin JM (1988) Synthesis of 2′-deoxy-2′,2′-difluoro-D-ribose and 2′-deoxy-2′,2′-difluoro-D-ribofuranosyl nucleosides. *J Org Chem* **53**: 2406–2409

Huang P, Chubb S, Hertel LW, Grindey GB and Plunkett W (1991) Action of 2′,2′-difluorodeoxycytidine on DNA synthesis. *Cancer Res* **51**: 6110–6117

Krakowski I, Petit T, Kayitalire L, Weber B, Beaudouin M, Canon JL, Janssens J, Martin C and Belpomme D (1998) Gemcitabine (Gemzar) in combination with cisplatin (CP) in advanced ovarian cancers (AOC): a phase II study. *Proc Am Soc Clin Oncol* **17**: 356a (abstract 1373)

Lu Y, Han J and Scanlon KJ (1988) Biochemical and molecular properties of cisplatin-resistant A2780 cells grown in folinic acid. *J Biol Chem* **263**: 4891–4894

Monks A, Scudiero D, Skehan P, Shoemaker R, Paull K, Vistica D, Hose C, Langley J, Cronise P, Vaigro-Wolff A, Gray-Goodrich M, Campbell H, Mayo J and Boyd M (1991) Feasibility of a high-flux anticancer drug screen using a diverse panel of cultured human tumor cell lines. *J Natl Cancer Inst* **83**: 761–766

Nogué M, Cirera L, Arcusa M, Tusquets I, Batiste-Alentorn E, Font A and Boto B (1998) Gemcitabine combined with cisplatin first line: a phase II study in

© 1999 Cancer Research Campaign 

patients with advanced epithelial ovarian cancer. *Proc Am Soc Clin Oncol* **17**: 357a (abstract 1377)

Parker RJ, Gill I, Tarone R, Vionnet J, Grunberg S, Muggia F and Reed E (1991) Platinum DNA-damage in leucocyte DNA of patients receiving cisplatin and carboplatin chemotherapy, measured by atomic absorption spectrometry. *Carcinogenesis* **12**: 1253–1258

Peters GJ, Laurensse E, Leyva A and Pinedo HM (1987) Purine nucleosides as cell-specific modulators of 5-fluorouracil metabolism and cytotoxicity. *Eur J Cancer Clin Oncol* **23**: 1869–1881

Peters GJ, Wets M, Keepers YPAM, Oskam R, Van Ark-Otte J, Noordhuis P, Smid K and Pinedo HM (1993*a*) Transformation of mouse fibroblasts with the oncogenes H-ras or trk is associated with pronounced changes in drug sensitivity and metabolism. *Int J Cancer* **54**: 450–455

Peters GJ, Schornagel JH and Milano GA (1993*b*) Clinical pharmacokinetics of anti-metabolites. *Cancer Surveys* **17**: 123–156

Ruiz van Haperen VWT, Veerman G, Vermorken JB and Peters GJ (1993) 2′,2′-Difluoro-deoxocytidine (Gemcitabine) incorporation into RNA and DNA of tumour cell lines. *Biochem Pharmacol* **46**: 762–766

Ruiz van Haperen VWT, Veerman G, Eriksson S, Boven E, Stegmann APA, Hermsen M, Vermorken JB, Pinedo HM and Peters GJ (1994*a*) Development and characterization of a 2′,2′-difluorodeoxycytidine-resistant variant of the human ovarian cancer cell line A2780. *Cancer Res* **54**: 4138–4143

Ruiz van Haperen VWT, Veerman G, Boven E, Noordhuis P, Vermorken JB and Peters GJ (1994*b*) Schedule-dependence of sensitivity to 2′,2′-difluorodeoxycytidine (Gemcitabine) in relation to accumulation and retention of its triphosphate in solid tumor cell lines and solid tumors. *Biochem Pharmacol* **48**: 1327–1339

Ruiz van Haperen VWT, Veerman G, Vermorken JB, Pinedo HM and Peters GJ (1996) Regulation of deoxycytidine kinase from solid tumor cell lines by CTP and UTP. *Biochem Pharmacol* **51**: 911–918

Scanlon KJ, Kashai-Sabet M, Tone T and Funato T (1991) Cisplatin resistance in human cancers. *Pharmacol Ther* **52**: 385–406

Steward WP, Dunlop DJ, Dabouis G, Lacroix H and Talbot D (1996) Phase I/II study of gemcitabine and cisplatin in non-small cell lung cancer: preliminary results. *Semin Oncol* **5**: 43–47

Sundquist WI and Lippard SJ (1990) The coordination chemistry of platinum anticancer drugs and related compounds with DNA. *Coord Chem Rev* **100**: 293–322

Terheggen PMAB, Emondt JY, Floot BGJ, Dijkman R, Schrier PI, Den Engelse L and Begg AC (1990) Correlation between cell killing by *cis*-diamminedichloroplatinum(II) in six mammalian cell lines and binding of a *cis*-diamminedichloroplatinum(II)-DNA antiserum. *Cancer Res* **50**: 3556–3561

Van der Vijgh WJF (1991) Clinical pharmacology of carboplatin. *Clin Pharmacokin* **21**: 242–261

Van der Wilt CL, Smid K, Noordhuis P, Aherne GW and Peters GJ (1997) Biochemical mechanisms of interferon modulation of 5-fluorouracil activity in colon cancer cells. *Eur J Cancer* **33**: 471–478

Van der Wilt CL, Visser GWM, Braakhuis BJM, Wedzinga R, Noordhuis P, Smid K and Peters GJ (1993) In vitro antitumour activity of cis- and trans-5-fluoro-5,6-dihydro-6-alkoxy-uracils. *Br J Cancer* **68**: 702–707

Van Moorsel CJA, Peters GJ and Pinedo HM (1997) Gemcitabine: future prospects of single-agent and combination studies. *Oncologist* **2**: 127–134

Van Moorsel CJA, Pinedo HM, Veerman G, Vermorken JB, Postmus PE and Peters GJ (1999) Scheduling of gemcitabine and cisplatin in Lewis Lung tumour bearing mice. *Eur J Cancer*. In press

Vermorken JB, Van der Vijgh WJF, Klein I, Gall HE, Van Groeningen CJ and Pinedo HM (1984) Pharmacokinetics of free and total platinum species after rapid and prolonged infusions of cisplatin. *Clin Pharmacol Ther* **39**: 136–144

*© 1999 Cancer Research Campaign*
